# Neoplastic plasma cells with concomitant azurophilic crystalline inclusions and Snapper‐Schneid bodies

**DOI:** 10.1002/jha2.1004

**Published:** 2024-09-03

**Authors:** Radu Chiriac, Luc‐Marie Gerland, Lucile Baseggio

**Affiliations:** ^1^ Laboratoire d'hématologie biologique, Hospices Civils de Lyon Pierre‐Benite France

**Keywords:** cytology, flow cytometry, multiple myeloma, plasma cell

1

A 66‐year‐old woman was being monitored for severe osteoporosis. Laboratory studies showed a 6 g/dL M spike and immunoglobulin M kappa paraprotein. Also, CRAB criteria were met.

The bone marrow (BM) aspirate revealed the presence of 30% atypical plasma cells (PC), which contained numerous large cytoplasmic azurophilic granules that appeared as dots resembling intracellular microorganisms. Concurrently, these cells were reminiscent of the “storage‐type” histiocytes reported in lysosomal storage diseases (Figure 1, Panels A and B; May‐Grunwald Giemsa stain [MGG], x100 objective). However, a subset of PC displayed coarse azurophilic granules, morphologically consistent with Snapper‐Schneid bodies (Figure 1, Panels A and B [arrows]; MGG stain, x100 objective). Flow cytometry of the BM aspirate revealed a monotypic CD38+/CD138+ PC population with an aberrant profile characterized by the loss of CD45 and CD19. These PC were also CD56‐, CD200+, CD20+, CD117+, and CD27‐ (Figure 1, Panel C), and expressed kappa immunoglobulin light chain, consistent with the observed paraprotein.

**FIGURE 1:**
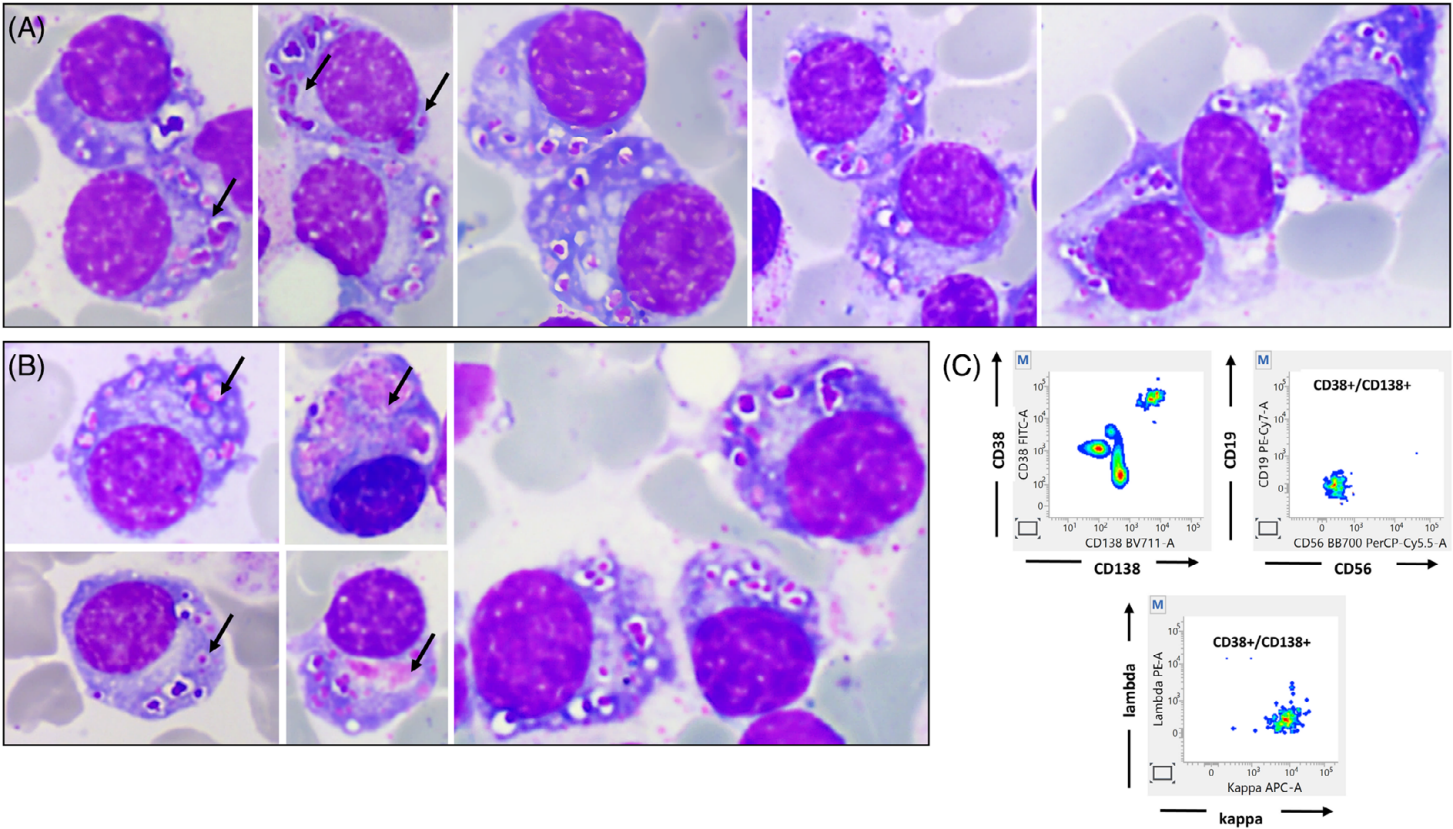
Panels A and B (x100 objective): Plasma cells with numerous large cytoplasmic granules and a few Snapper‐Schneid bodies (arrows). Panel C: Aberrant flow cytometry profile of plasma cells—CD38+/CD138+, loss of CD45, CD19, and CD56, expressing kappa immunoglobulin light chain.

Various types of cytoplasmic inclusions have been documented in plasma cell neoplasms, including Russell bodies, crystals, and Auer rod‐like inclusions, while azurophilic granules and Snapper‐Schneid bodies remain uncommon. This myeloma case is particularly noteworthy as it demonstrates the presence of both azurophilic granules and Snapper‐Schneid bodies in an untreated patient with a rare paraprotein: immunoglobulin M kappa. Previous case reports have documented this phenomenon in pretreated patients, including those involving diamidine treatment [1].

The presence of PC with atypical granules and an aberrant immunophenotype underscores diagnostic complexity, requiring thorough morphological and immunological examination. Careful distinction of these features from those of microbial infections or lysosomal storage diseases is essential to ensure appropriate clinical management.

## AUTHOR CONTRIBUTIONS

Radu Chiriac wrote the manuscript; Lucile Baseggio and Luc‐Marie Gerland conducted the cytological and flow cytometric studies. All authors contributed to the final manuscript.

## CONFLICT OF INTEREST STATEMENT

The authors declare no conflict of interest.

## FUNDING INFORMATION

The authors received no specific funding for this work.

## ETHICS STATEMENT

This manuscript respects the ethical policy of Hospices Civils de Lyon for the treatment of human research participants.

## PATIENT CONSENT STATEMENT

No patient‐identifying data were used. The authors did not obtain written informed consent from the patient but the patient did not object to his data being used for research purposes (as required by the ethics policy of Hospices Civils de Lyon).

## CLINICAL TRIAL REGISTRATION

The authors have confirmed clinical trial registration is not needed for this submission.

## Data Availability

Data sharing is not applicable to this article as no new data were created or analyzed in this study.

## References

[1] Snapper I , Schneid B . On the influence of stilbamidine upon myeloma cells. Blood. 1946;1(6):534–536. 21002321

